# Prostate-specific membrane antigen in malignant brain tumors: vascular heterogeneity, imaging promise, and therapeutic challenges

**DOI:** 10.3389/fonc.2026.1934007

**Published:** 2026-09-08

**Authors:** Bihani Pokharel, Mingtao Feng, Yuechao Yang, Zhe Qi, Sabrina Shah Kazmi, Yiqun Cao, Liangdong Li, Yang Gao

**Affiliations:** 1 Department of Neurosurgery, Fudan University Shanghai Cancer Center, Shanghai, China; 2 Department of Oncology, Shanghai Medical College, Fudan University, Shanghai, China

**Keywords:** brain metastases, gliomas, molecular imaging, prostate-specific membrane antigen, radioligand therapy

## Abstract

Malignant brain tumors have high morbidity and poor prognosis. Prostate-specific membrane antigen (PSMA) has emerged as a promising vascular biomarker in this setting, particularly in gliomas and brain metastases. This review synthesizes current evidence on PSMA expression patterns, regulatory mechanisms, imaging applications, and translational potential in malignant brain tumors. Across studies, PSMA expression in gliomas is predominantly localized to tumor-associated neovasculature and closely associated with angiogenic signaling. In brain metastases, PSMA expression appears more heterogeneous and more strongly influenced by metastatic origin and the brain microenvironment. These biological features provide a rationale for PSMA-targeted positron emission tomography, which offers high lesion-to-background contrast. Clinically, PSMA-targeted imaging shows promise for glioma grading, selected post-treatment assessment, and detection of brain metastases across multiple primary tumors. However, therapeutic translation remains limited because compartment-specific target distribution, inconsistent target retention, delivery barriers, and uncertain dosimetry continue to constrain the efficacy of PSMA-targeted treatment strategies in malignant brain tumors. Current evidence supports PSMA as a promising complementary imaging biomarker rather than as a validated therapeutic target in malignant brain tumors. Future progress will require standardized detection methods, stronger mechanistic validation, and dosimetry-guided clinical development.

## 1 Introduction

Gliomas and brain metastases (BM) are among the most common malignant tumors of the central nervous system (CNS). Among gliomas, glioblastoma (GBM) is the most aggressive primary brain tumor in adults, with a median overall survival of only 12–15 months despite multiple therapeutic approaches (1–5). BM, which most frequently arises from lung, breast, melanoma, renal cell carcinoma, and colorectal primaries, affects about 10–40% of patients with advanced solid tumors and represents a major burden on cancer care (6, 7). Despite these differences, both gliomas and BM are characterized by prominent tumor-associated angiogenesis, making vascular biomarkers and vascularly targetable pathways particularly relevant in malignant brain tumors (8, 9). This shared vascular dependency makes malignant brain tumors a useful setting for evaluating whether vascularly expressed biomarkers can be translated into clinically meaningful imaging and therapeutic strategies.

Prostate-specific membrane antigen (PSMA), also known as glutamate carboxypeptidase II and encoded by the folate hydrolase 1 (*FOLH1*) gene, is a type II transmembrane glycoprotein with well-described enzymatic functions, including folate hydrolase and neuropeptidase activity (10–13). Its neuropeptidase activity also gives PSMA physiological relevance in the CNS, indicating that its interpretation in brain tumors requires a biological context rather than a purely oncologic framework (11). PSMA first gained clinical importance because of its strong and selective expression in prostate cancer, where it became an important target for molecular imaging and radioligand therapy (14). This success established PSMA as a major theranostic target in oncology and stimulated broader interest in its expression beyond prostate malignancy.

Subsequent studies showed that PSMA is not restricted to prostate cancer epithelium. It is also expressed in the neovasculature of multiple non-prostatic solid tumors, including malignant brain tumors in the CNS (15, 16). This selective vascular expression has expanded interest from PSMA as a prostate cancer biomarker to PSMA as a broader theranostic target in neuro-oncology. In malignant brain tumors, PSMA expression has been most consistently detected in tumor-associated vascular compartments rather than in normal brain vasculature, providing a biologically attractive basis for high-contrast molecular imaging (17, 18). The therapeutic implications of this predominantly vascular localization are likely to be modality-dependent. Although vascular localization may limit retention or dose delivery for some radioligand approaches, endothelial PSMA may remain accessible to circulating antibody- or immune-based strategies. At the same time, emerging evidence suggests that PSMA biology in malignant brain tumors is more complex than a simple vascular marker model. Its expression patterns appear to differ between gliomas and BM, and accumulating data suggest that microenvironmental regulation, spatial compartmentalization, and tumor-type heterogeneity may influence how PSMA should be interpreted in neuro-oncology (16, 19).

These observations have positioned PSMA as a promising biomarker and potential therapeutic target in malignant brain tumors, though its therapeutic feasibility remains to be established. However, critical uncertainties remain regarding where PSMA is expressed, how it is regulated, how reliably imaging reflects the biologically meaningful presence of the target, and whether current therapeutic approaches can deliver sufficient intracranial benefit. This narrative review integrates current evidence on PSMA, with emphasis on its expression, biological regulation, imaging applications, and translational potential in malignant brain tumors. Particular emphasis is placed on emerging evidence that PSMA regulation differs between primary gliomas and BM, reflecting distinct microenvironmental influences and cellular sources of expression in each setting.

### 1.1 Literature search methodology

This article was structured as a narrative review rather than a systematic or scoping review. A targeted literature search was conducted primarily using PubMed/MEDLINE, covering publications from database inception to March 2026. Search terms included combinations of “prostate-specific membrane antigen,” “PSMA,” “*FOLH1*,” “glioma,” “glioblastoma,” “brain metastasis,” “brain metastases,” “angiogenesis,” “neovasculature,” “positron emission tomography,” “PET,” “molecular imaging,” “radioligand therapy,” “theranostics,” and “PSMA-targeted therapy.” English-language studies directly addressing PSMA expression, cellular localization, regulatory mechanisms, molecular imaging, or therapeutic applications in malignant brain tumors were prioritized. Peer-reviewed original studies, prospective and retrospective clinical studies, systematic reviews and meta-analyses, and mechanistic or translational investigations were preferentially included. Because clinical evidence for PSMA-targeted therapy in malignant brain tumors remains limited, selected case reports, small clinical series, conference abstracts, and preclinical studies were also considered when they provided relevant information on therapeutic feasibility, delivery, dosimetry, or emerging treatment strategies. Seminal studies defining PSMA biology were retained irrespective of publication date. Given the heterogeneous evidence base, study design and clinical maturity were explicitly considered in the narrative synthesis to distinguish established clinical observations from preliminary or preclinical findings. Reference lists of relevant primary studies and reviews were also screened to identify additional pertinent publications. As a narrative review, formal PRISMA-based screening, risk-of-bias assessment, and quantitative meta-analysis were not performed; the aim was critical integration of biological, imaging, and translational evidence rather than exhaustive aggregation of evidence.

## 2 Prostate-specific membrane antigen expression and mechanisms in malignant brain tumors

### 2.1 Prostate-specific membrane antigen expression and mechanisms in gliomas

#### 2.1.1 Prostate-specific membrane antigen expression in gliomas

In the CNS, PSMA exhibits a highly restricted expression pattern, with minimal to absent protein detectable in normal brain parenchyma and its associated vasculature. Early studies established that PSMA is expressed in the neovasculature of multiple solid tumors but is largely absent from normal vessels, a finding consistently confirmed across different antibody clones and methodological approaches (17, 20, 21). In glioma, Wernicke et al. first demonstrated vascular PSMA expression in all GBM specimens examined, whereas adjacent normal brain tissue remained negative (18). This observation provided an important biological basis for considering PSMA as a selective imaging biomarker and candidate therapeutic target in glioma.

Evidence for PSMA expression in gliomas can be organized across four complementary domains: histopathology, molecular imaging, transcriptomic and biopsy-correlative studies, and clinical outcome analyses. At the histopathological level, subsequent immunohistochemical studies demonstrated that PSMA expression in gliomas is strongly associated with tumor grade. In the largest treatment-naive series, vascular PSMA expression was identified in 26 of 122 glioma specimens and was highly enriched in high-grade tumors: 81% of PSMA-positive cases were GBM, compared with 10% of WHO grade III gliomas and 2% of grade II gliomas (*p* < 0.001) (22). Independent studies have likewise confirmed vascular PSMA positivity in a substantial proportion of GBM specimens (23). In addition to its association with histological grade, vascular PSMA expression has been reported more frequently in tumors lacking detectable IDH1-R132H mutation, linking PSMA to a more aggressive molecular phenotype (22). Because several studies in the PSMA literature predate the 2021 WHO classification or report gliomas according to histological grade, the terms low-grade glioma (LGG) and high-grade glioma (HGG) are retained where they reflect the terminology and cohort definitions used in the original studies.

At the molecular imaging level, PSMA-targeted PET findings broadly parallel these histopathological observations. Treatment-naive high-grade gliomas (HGG) showed markedly higher ^68^Ga-PSMA-11 positron emission tomography/computed tomography (PET/CT) uptake than low-grade gliomas (LGG), with reported mean SUVmax values of 16.93 ± 5.4 vs. 2.93 ± 0.30 and tumor-to-background ratios (TBR) of 13.95 vs. 3.42 (24). Significant correlations have also been reported between PSMA uptake and glioma grade (*r* = 0.883, *p* < 0.001), Ki-67 proliferation index, and IDH-wildtype status (25). Similar patterns have been observed using other PSMA-targeted tracers, including ^18^F-DCFPyL, ^18^F-PSMA-1007, and [^99m^Tc]Tc-iPSMA single-photon emission computed tomography (SPECT), with uptake predominantly localized to HGG or recurrent lesions and largely absent in treatment-naive LGG and reactive gliosis (26–28). Exploratory studies using ^89^Zr-Df-IAB2M anti-PSMA minibodies further suggest a relationship between tracer uptake and tissue-level PSMA expression (29). These findings suggest that PSMA-targeted PET uptake reflects, in part, the angiogenic activity and biological aggressiveness of HGG.

At the transcriptomic and biopsy-correlative level, however, important discrepancies indicate that the relationship between imaging signal and tissue-level PSMA expression is more complex than suggested by histology and PET studies alone. A multicenter inventory study found no significant correlation between PSMA PET radioligand uptake and immunohistochemical PSMA expression in either endothelial or tumor cells, indicating that tracer accumulation may be influenced by vascular permeability, perfusion, and blood–brain barrier disruption rather than target abundance alone (19, 30). This complexity is further supported by large-scale transcriptomic analyses showing that overall *FOLH1* expression in CNS tumors may be lower than in normal brain tissue, highlighting the importance of distinguishing between bulk transcript levels and compartment-specific vascular protein expression (16). In contrast, a prospective PET–magnetic resonance imaging (MRI)-guided multiregional biopsy study demonstrated that PSMA PET uptake correlates more closely with microvascular PSMA expression than with tumor-cell expression (*r* = 0.487, *p* < 0.01), reinforcing the interpretation that imaging signals predominantly reflect tumor-associated neovasculature (31). Taken together, these findings indicate that the PSMA PET signal is shaped by both target expression and microenvironmental factors and should be interpreted as a vascular and context-dependent biomarker rather than a direct surrogate of tumor-cell PSMA expression.

From a clinical perspective, the prognostic significance of PSMA expression in glioma remains uncertain. Yuile et al. reported that higher vascular PSMA expression in IDH-wildtype GBM was associated with longer overall survival on univariate analysis, although this association did not remain significant after multivariable adjustment (32). In contrast, other studies have suggested associations between vascular PSMA expression and poorer outcomes, including shorter survival or worse post-recurrence prognosis, although these findings have not been consistently significant across cohorts (22, 33). These inconsistencies likely reflect differences in cohort composition, treatment status, timing of assessment, and quantification methodologies. Accordingly, while PSMA represents a robust marker of glioma-associated neovasculature, its prognostic value remains to be clarified in larger, well-standardized studies. From a translational perspective, these findings support PSMA as a vascular imaging biomarker in glioma, while emphasizing that vascular or tumor-cell PSMA expression, PET tracer uptake, and therapeutic targetability are related but non-equivalent parameters.

#### 2.1.2 Mechanisms of prostate-specific membrane antigen in gliomas

In glioma, accumulating evidence suggests that PSMA is not merely a passive vascular marker but may also participate actively in tumor-associated angiogenesis. Consistent with its spatial distribution, PSMA expression in GBM is predominantly localized to the neovascular compartment and appears to be shaped by microenvironmental signals rather than uniform tumor-cell expression. Current mechanistic evidence in gliomas can be understood in terms of upstream induction of PSMA expression, downstream endothelial signaling, and integration with broader angiogenic pathways. This distinction situates PSMA within the biology of the glioma vascular niche, where endothelial activation, hypoxia, and stromal interactions are central to disease progression (16, 18, 23, 31, 34).

One of the most clearly defined upstream regulatory mechanisms is the SPP1–HIF-1α axis. Tu et al. demonstrated that glioma-derived secreted phosphoprotein 1 (SPP1, osteopontin) induces PSMA expression in endothelial cells through HIF1-α–dependent transcriptional regulation. In this model, HIF-1α knockdown attenuated PSMA expression and suppressed endothelial migration and tube formation, supporting a mechanism in which hypoxia-associated tumor signals drive vascular PSMA upregulation as part of a pro-angiogenic response (35). These findings are consistent with broader observations that vascular PSMA expression correlates with tumor grade and hypoxia-associated microenvironmental features in gliomas (22, 36).

Beyond its regulation, PSMA has been shown to exert functional effects on angiogenic signaling. Gao et al. reported that PSMA enhances endothelial proliferation, migration, invasion, and tube formation, while pharmacological inhibition using 2-(phosphonomethyl)pentanedioic acid (2-PMPA) attenuates these processes. Mechanistically, PSMA was shown to interact with integrin β4 (ITGB4) and activate NF-κB–related signaling pathways, leading to increased expression of pro-angiogenic and inflammatory mediators, including interleukin 6 (*IL6*), tumor necrosis factor (*TNF*), C-X-C motif chemokine ligand 1 (*CXCL1*), endothelin-1 (*EDN1*), and intercellular adhesion molecule 1 (*ICAM1*). *In vivo*, PSMA inhibition reduced tumor growth and was associated with decreased ITGB4 expression and NF-κB activation, supporting a functional role for PSMA-associated signaling in promoting angiogenesis within the tumor microenvironment (37). Although direct glioma-specific validation of this axis remains limited, these findings are consistent with broader evidence that PSMA modulates integrin-dependent endothelial signaling during pathological angiogenesis (38, 39).

Recent studies further suggest that PSMA-associated signaling may intersect with canonical VEGF-driven angiogenic pathways in GBM. In a large cohort of IDH-wildtype GBM, vascular PSMA expression was positively correlated with VEGF levels (32), a finding also observed in independent studies (33, 36). Given that NF-κB activation downstream of PSMA-ITGB4 signaling can promote VEGF expression (37), this relationship may reflect coordinated activation of multiple pro-angiogenic pathways within tumor-associated endothelium. However, whether PSMA acts upstream of VEGF signaling, operates in parallel, or represents a compensatory pathway remains to be clarified.

**Figure 1:**
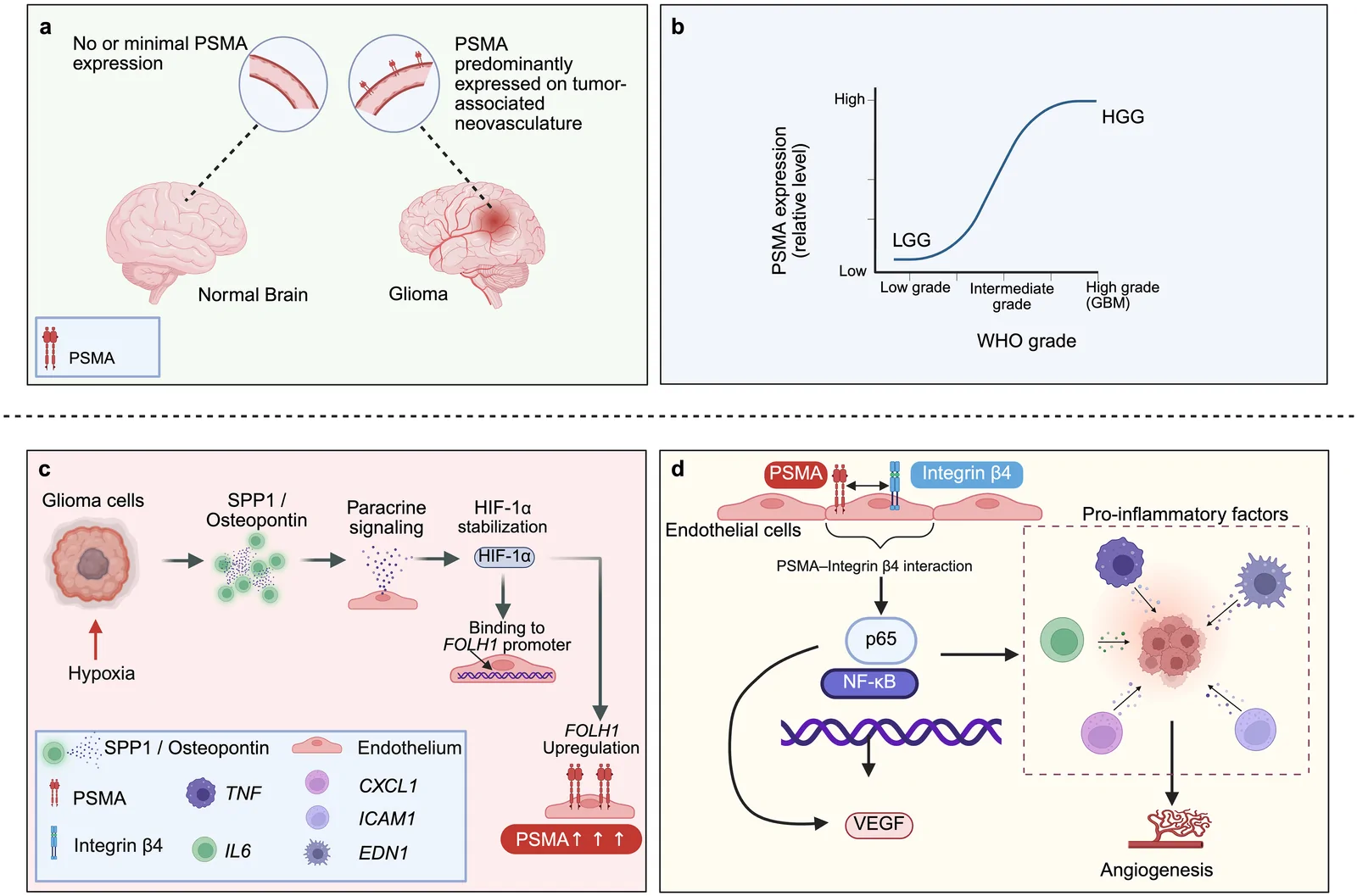
Expression and mechanisms of PSMA in gliomas. **(a)** PSMA is minimal to absent in normal brain vasculature but is predominantly expressed in tumor-associated neovasculature in glioma. **(b)** Vascular PSMA expression increases with WHO grade, from low-grade glioma (LGG) to high-grade glioma (HGG), particularly glioblastoma (GBM). **(c)** Under hypoxic conditions, glioma-derived SPP1/osteopontin promotes HIF-1α-dependent transcriptional upregulation of *FOLH1*/PSMA in endothelial cells. **(d)** PSMA–integrin β4 interaction in glioma endothelium activates p65/NF-κB signaling, inducing transcriptional upregulation of inflammatory and angiogenic genes, including *TNF*, *IL6*, *CXCL1*, *ICAM1*, and *EDN1*, and contributing to VEGF-associated angiogenic signaling. Created with BioRender.com. Pokharel, B. (2026), https://BioRender.com/2c8tbav.

Taken together, current evidence supports a model in which PSMA in gliomas functions as a microenvironment-responsive, predominantly vascular mediator of angiogenesis. Upstream glioma-derived signals such as SPP1 induce PSMA expression through hypoxia-related pathways, while downstream PSMA activity promotes endothelial activation via ITGB4 and NF-κB–associated signaling and may interact with VEGF-dependent mechanisms. At the same time, transcriptomic and biopsy-correlative studies caution against extrapolating PSMA biology from bulk tumor measurements, as *FOLH1* expression at the transcript level may not reflect compartment-specific protein localization, and imaging signals primarily track vascular PSMA rather than tumor-cell expression (16, 31, 40). Therefore, PSMA in glioma is best understood as a compartment-specific vascular effector that integrates microenvironmental signals to regulate tumor angiogenesis, rather than as a tumor-cell–intrinsic driver (**Figure 1**). This compartment-specific mechanism provides a biological rationale for PSMA-targeted imaging of angiogenic tumor vasculature but also explains why therapeutic translation may require delivery and dosimetry strategies that account for vascular rather than tumor-cell–intrinsic target localization.

### 2.2 Prostate-specific membrane antigen expression and mechanisms in brain metastases

#### 2.2.1 Prostate-specific membrane antigen expression in brain metastases

PSMA expression has been reported across multiple BM subtypes, but unlike the relatively consistent vascular pattern described in GBM, its distribution in BM appears more heterogeneous across primary tumor types and may involve both vascular and non-vascular compartments. Current evidence nevertheless supports the view that PSMA is most commonly localized to tumor-associated neovasculature, with marked differences in staining intensity, frequency, and possible cellular sources depending on the metastatic origin (41–49). Because PSMA expression in BM varies substantially by metastatic origin, the available evidence is most clearly interpreted by primary tumor type.

Lung cancer is one of the most common primary sources of BM, making lung cancer brain metastases (LCBM) a particularly important subtype for evaluating PSMA biology (50). Among BM subtypes, LCBM have shown particularly strong and consistent PSMA expression. In a paired cohort of primary lung carcinomas and matched BM, vascular PSMA positivity was observed in all BM specimens, with staining intensity greater than that observed in breast cancer and melanoma BM (41). In the same study, vascular PSMA expression in the primary lung tumor was associated with more rapid dissemination to the brain, whereas tumor-cell PSMA expression was not, suggesting that vascular PSMA may be linked to metastatic behavior rather than simply reflecting established intracranial disease (41). Complementing these observations, Pei et al. reported significantly higher PSMA uptake in lung adenocarcinoma BM than in matched primary lung tumors on ^68^Ga-PSMA PET/CT, and single-cell analysis suggested enrichment of *FOLH1* expression in oligodendrocyte-lineage cells within the metastatic brain microenvironment (42). More recently, a prospective PET–MRI-guided multiregional biopsy study showed that strong PSMA expression in BM was most commonly microvascular, whereas tumor-cell PSMA expression was identified only in a minority of BM samples and was not strongly expressed; notably, PSMA positivity was also detected in non-malignant glial cells within tumor tissue (31). This distinction is translationally important because PSMA PET uptake in LCBM may reflect a combined vascular and brain-niche signal rather than a single tumor-cell target compartment.

Breast cancer is also a major primary source of BM (51). Available evidence indicates that breast cancer BM frequently demonstrates PSMA expression, most reproducibly within tumor-associated vasculature. In a pilot study of breast cancer BM, vascular PSMA staining was consistently observed, although staining intensity was generally lower than in matched primary breast tumors (45). Broader breast cancer studies have confirmed prominent PSMA expression in tumor-associated vasculature and variability across metastatic lesions (43–45).

Melanoma BM also exhibits PSMA-positive neovasculature in available series, although the reported staining intensity appears lower than that observed in LCBM (41). This suggests that PSMA may still serve as a vascular marker in melanoma BM, but with less robust expression than in some other metastatic subtypes. At present, however, mechanistic and clinicopathological data in melanoma BM remain limited.

Thyroid cancer BM, particularly in radioiodine-refractory disease, may demonstrate very high PSMA tracer uptake. In a study of well-differentiated metastatic thyroid cancer, BM from papillary thyroid carcinoma showed striking uptake on PSMA-targeted imaging, with lesion avidity exceeding that of other metastatic sites (46). Subsequent case reports and small series have supported the feasibility of PSMA imaging in thyroid cancer BM, particularly when conventional radioiodine-based approaches are limited or negative (46–48). Although these findings are clinically intriguing, the biological basis for this high uptake remains insufficiently characterized, and systematic tissue-based validation in thyroid BM is still sparse.

For renal cell carcinoma (RCC) BM, evidence remains particularly limited and is derived mainly from case-based PSMA PET observations rather than dedicated tissue studies; therefore, the frequency and cellular localization of PSMA expression in RCC BM remain poorly defined (49). An important exception is prostate cancer BM, in which PSMA avidity may reflect tumor-cell expression rather than the predominantly vascular pattern described in most non-prostatic BM, although dedicated tissue-level characterization remains limited (52, 53).

**Figure 2:**
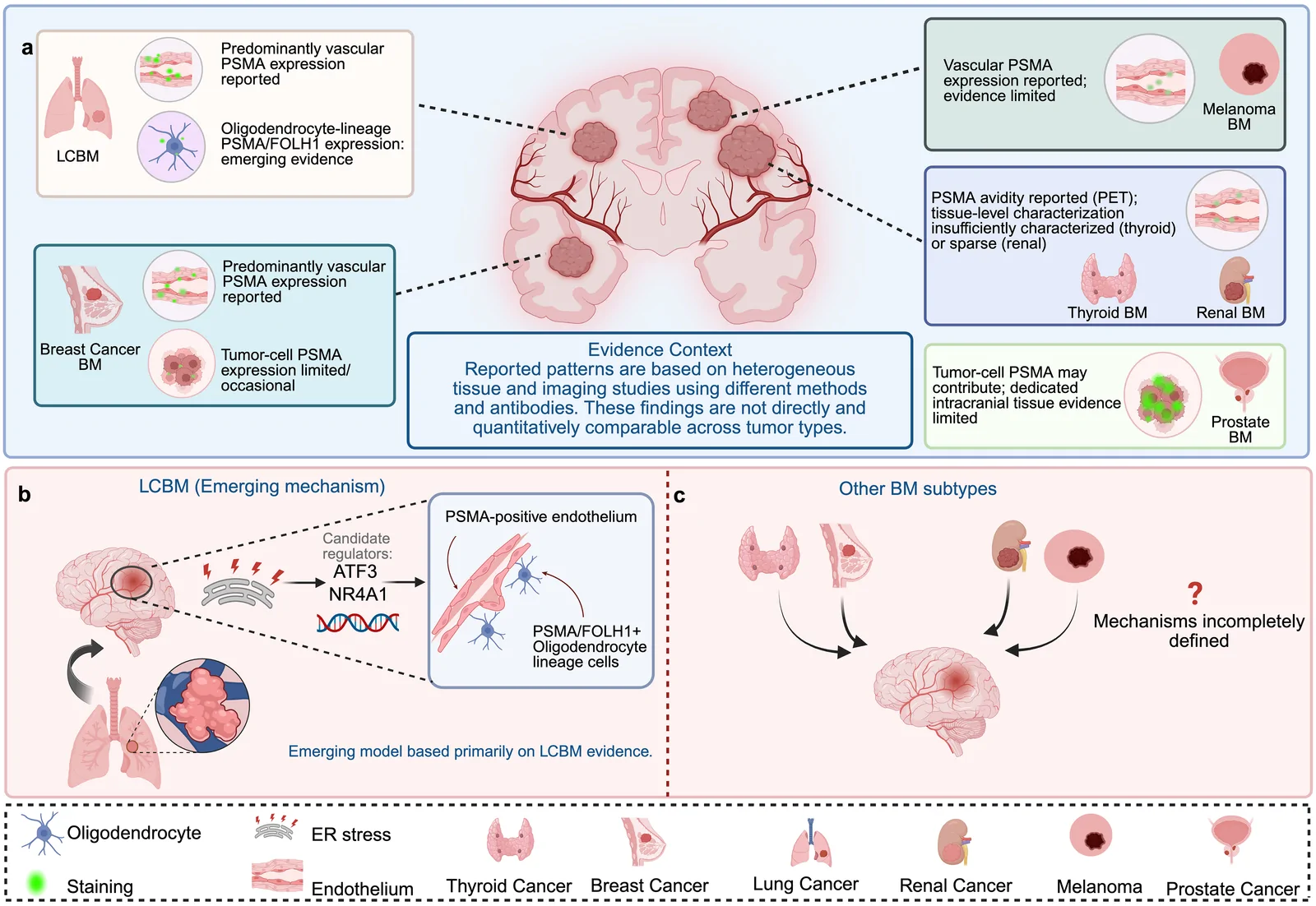
Expression and mechanisms of PSMA in brain metastases. **(a)** PSMA expression in brain metastases (BM) varies according to primary tumor origin and cellular compartment. Lung cancer BM (LCBM) show predominantly vascular PSMA expression with emerging evidence of oligodendrocyte-lineage PSMA/*FOLH1* expression. Breast cancer BM show predominantly vascular expression, whereas evidence in melanoma BM remains limited. PSMA avidity has been reported in thyroid and renal BM, although tissue-level characterization remains limited or sparse. In prostate cancer BM, tumor-cell PSMA may contribute, but dedicated intracranial tissue evidence remains limited. **(b)** In LCBM, ER stress-associated signaling involving ATF3 and NR4A1 has been implicated in PSMA/*FOLH1* regulation in PSMA-positive endothelium and oligodendrocyte-lineage cells, representing an emerging mechanistic model based primarily on LCBM evidence. **(c)** In contrast, the mechanisms driving PSMA expression in other BM subtypes remain incompletely defined. Created with BioRender.com. Pokharel, B. (2026), https://BioRender.com/96doq1h.

Taken together, available evidence indicates that PSMA biology in BM is heterogeneous and tumor-type-dependent. LCBM show the most consistent and biologically distinctive pattern reported to date, combining strong vascular expression with emerging evidence for microenvironment-associated non-endothelial contribution. Breast cancer BM are supported by tissue-based evidence of predominantly vascular PSMA expression, whereas evidence in melanoma remains limited. In thyroid and renal BM, the literature is more heavily derived from imaging studies, small series, and case reports, with sparse tissue-level characterization. Prostate cancer BM represent an uncommon but distinct setting in which tracer avidity may more directly reflect tumor-cell PSMA, although dedicated intracranial tissue evidence remains limited. Overall, these findings suggest that PSMA in BM should not be viewed as a uniform biomarker, but rather as a heterogeneous target whose spatial distribution and biological interpretation depend on metastatic context (**Figure 2a**). From a translational perspective, this heterogeneity supports tumor-type-specific use of PSMA PET and cautions against generalizing PSMA-directed therapeutic strategies across all BM without compartment-specific validation. Accordingly, PSMA PET avidity in BM should not be assumed to reflect tumor-cell PSMA abundance or therapeutic targetability, particularly when the imaging signal may arise predominantly from tumor-associated vasculature.

#### 2.2.2 Mechanisms of prostate-specific membrane antigen in brain metastases

Compared with glioma, where PSMA regulation has been linked to defined angiogenic pathways, the mechanistic basis of PSMA expression in BM remains less well characterized and appears to be more strongly shaped by metastatic context. Current evidence suggests that PSMA biology in BM is more heterogeneous, with differences in primary tumor origin, brain microenvironment, and cellular compartment all likely contributing to its expression pattern. Although PSMA is most consistently detected in tumor-associated neovasculature across several BM subtypes, emerging data indicate that its regulation in at least some metastases may extend beyond a purely endothelial model.

The clearest mechanistic insights to date come from LCBM. In this setting, single-cell RNA sequencing and imaging-pathology analyses suggest that *FOLH1* expression may be enriched not only in vascular compartments but also in oligodendrocyte-lineage cells within the metastatic brain microenvironment (42). Pei et al. further identified activating transcription factor 3 (ATF3) and nuclear receptor subfamily 4 group A member 1 (NR4A1) as candidate upstream regulators of PSMA expression, implicating stress-responsive transcriptional programs in this process (**Figure 2b**) (42). Gene set analyses in the same study linked PSMA-associated expression patterns to endoplasmic reticulum stress, unfolded protein response pathways, and protein processing in the endoplasmic reticulum, suggesting that PSMA upregulation in LCBM may reflect microenvironment-driven cellular stress rather than the predominantly hypoxia-associated vascular signaling described in glioma. More recently, biopsy-correlative data showed that BM PSMA expression is usually strongest in microvasculature, while strong tumor-cell expression is uncommon and PSMA positivity can also be found on non-malignant glial cells, reinforcing the possibility that BM PSMA reflects both vascular and microenvironment-associated components (31). These findings support the possibility that the metastatic brain niche shapes PSMA expression through local stromal or glial responses.

In addition to its intracranial regulation, PSMA may also be linked to the metastatic behavior of the primary tumor. In a paired study of lung carcinomas and matched BM, vascular PSMA expression in the primary lung tumor was associated with faster dissemination to the brain, whereas tumor-cell PSMA expression was not (41). Although this does not establish a mechanism directly, it raises the possibility that PSMA-positive tumor vasculature may mark or contribute to a pro-metastatic phenotype. Potential explanations include enhanced angiogenic signaling, altered endothelial permeability, or vascular conditions favorable to tumor cell intravasation and later brain colonization. At present, however, these possibilities remain inferential, and direct functional validation is still lacking.

For other BM subtypes, mechanistic understanding remains limited. Breast cancer, melanoma, and thyroid cancer BM frequently exhibit vascular PSMA expression, but the regulatory pathways responsible for this pattern have not been well characterized (41, 43–48). It remains unclear whether PSMA in these lesions is driven mainly by general angiogenic programs, by brain-specific stromal interactions, or by molecular features inherited from the primary tumor. Likewise, whether PSMA contributes functionally to metastatic progression, vascular remodeling, or therapeutic resistance in BM has not yet been established. In contrast to glioma, where PSMA has been experimentally linked to endothelial signaling and angiogenesis, evidence in BM remains largely descriptive.

Taken together, current data suggest that PSMA in BM should be understood as a context-dependent marker whose regulation differs from that in primary glioma. In LCBM, the most compelling model is one of brain microenvironment-associated induction involving stress-responsive transcriptional programs and possible oligodendrocyte-lineage participation. In other BM subtypes, PSMA is most consistently observed in tumor-associated vasculature, but its upstream drivers and downstream functional significance remain uncertain. Importantly, the mechanistic evidence for PSMA regulation in BM remains largely inferential and is driven primarily by a single mechanistic study in LCBM (42). Whether the ATF3/NR4A1/ER stress axis operates in other BM subtypes, or whether alternative pathways contribute, has not been investigated. Experimental validation using *in vitro* or *in vivo* models of BM is urgently needed to establish causality and to determine whether PSMA in BM represents an actionable therapeutic target or primarily a correlative biomarker. Therefore, the most defensible conclusion at present is that PSMA in BM represents a biologically heterogeneous target, with regulatory mechanisms that remain incompletely resolved and are likely to vary according to metastatic origin and intracranial niche (**Figure 2**). This uncertainty has direct translational implications. PSMA PET and PSMA-directed interventions in BM should be interpreted and developed in a tumor-type-specific manner, with tissue-level or imaging-pathology validation required before therapeutic strategies are extrapolated across metastatic subtypes.

## 3 Prostate-specific membrane antigen-targeted imaging in malignant brain tumors

### 3.1 Prostate-specific membrane antigen positron emission tomography imaging in gliomas

PSMA-targeted PET has been investigated as a promising molecular imaging approach in glioma because of its low physiological background uptake in normal brain parenchyma and the preferential localization of PSMA in tumor-associated neovasculature. This combination provides a high lesion-to-background contrast and creates a biologically attractive framework for intracranial imaging. In treatment-naive cohorts, HGG consistently show substantially higher PSMA tracer uptake than low-grade tumors, and several studies using ^68^Ga-PSMA-11 PET/CT have reported significant associations between PSMA PET parameters and established markers of tumor aggressiveness, including histological grade, Ki-67 proliferation index, and IDH-wildtype status (24, 25). These findings suggest that PSMA PET may capture, at least in part, the angiogenic activity and biological aggressiveness of glioma.

The diagnostic value of PSMA PET in glioma has been supported by studies using multiple tracers, including ^68^Ga-PSMA-11 PET/CT, ^18^F-DCFPyL PET, ^18^F-PSMA-1007 PET/CT, and [^99^mTc]Tc-iPSMA SPECT (24–28). Across these studies, uptake is generally strongest in HGG and recurrent lesions, whereas treatment-naive LGG and reactive gliosis show little or no avidity. Comparative analyses have suggested that PSMA PET may offer advantages over ^18^F-fluorodeoxyglucose (FDG) PET/CT for glioma evaluation, particularly for lesion conspicuity and tumor delineation (54). A meta-analysis of diagnostic studies likewise reported favorable pooled performance of PSMA-targeted PET for differentiating HGG from LGG, although cohort sizes remain limited and methodological heterogeneity is substantial (40). Accordingly, current evidence supports PSMA PET as a potentially useful adjunct for glioma grading and lesion characterization, but not yet as a replacement for established imaging standards.

An important limitation is that tracer uptake does not necessarily correspond directly to total tumor *FOLH1* expression or uniform target abundance. Rather, imaging signal appears to reflect compartment-specific vascular expression, local perfusion, blood–brain barrier disruption, and tracer accessibility (30, 31). This is particularly relevant in glioma, where a recent PET–MRI-guided multiregional biopsy study indicated that PSMA PET uptake tracks more closely with microvascular PSMA expression than with tumor-cell expression (31). Therefore, PSMA PET should be interpreted primarily as an imaging marker of tumor-associated vascular biology rather than as a simple readout of whole-tumor PSMA expression.

### 3.2 Prostate-specific membrane antigen positron emission tomography imaging in brain metastases

PSMA PET has also been investigated as a promising imaging approach in BM. Available evidence indicates that PSMA-avid BM can be detected across multiple primary tumor types, including lung cancer, breast cancer, melanoma, thyroid carcinoma, salivary gland tumors, renal cell carcinoma, and prostate cancer (42, 46–49, 52, 53, 55). In systematic review data, PSMA-targeted PET detected the large majority of reported BM lesions and, in some cases, identified metastases that were poorly visualized or missed on ^18^F-FDG PET because of the inherent limitations of glucose-based imaging in the brain (49). A recent prospective study of 27 patients with BM from diverse primary tumors further demonstrated PSMA-positive lesions in 26 patients and identified additional lesions not detected on ^18^F-FDG PET/CT in 42% of cases, supporting the potential added diagnostic value of PSMA-targeted imaging in selected intracranial settings (56).

Among metastatic subtypes, LCBM have been studied most directly. In this setting, ^68^Ga-PSMA PET/CT demonstrated significantly higher uptake in BM than in matched primary lung tumors, supporting the concept that the metastatic brain environment may amplify the PSMA-related imaging signal (42). Prostate cancer BM have also been well characterized with PSMA-targeted imaging, although the available evidence is derived mainly from incidental-lesion series and dural-lesion characterization studies rather than dedicated BM cohorts. Because BM are rare in prostate cancer, current data remain limited but suggest that PSMA PET may be useful for identifying and characterizing selected intracranial lesions in this setting (52, 53). Thyroid cancer BM, particularly in radioiodine-refractory disease, may show especially intense PSMA uptake, with some reports describing very high lesion avidity and favorable contrast relative to other metastatic sites (46–48). Case-based and small cohort data also support detectability in melanoma and renal cell carcinoma BM, although the evidence base remains much thinner for these tumor types (49). Overall, the current literature suggests that PSMA PET may be particularly useful in BM from selected primaries with strong vascular or microenvironment-associated PSMA expression, but prospective comparative studies with MRI and non-PSMA PET tracers remain limited.

Despite this promise, the clinical role of PSMA PET in BM remains incompletely defined. In routine practice, contrast-enhanced MRI remains the primary imaging modality for diagnosis and follow-up, and most BM are already well visualized on MRI. Therefore, the added value of PSMA PET is likely to lie not in simple lesion detection alone, but in selected contexts such as biological characterization, problem solving when conventional imaging is equivocal, or treatment selection for PSMA-directed approaches, or potentially for identifying lesions with particularly favorable theranostic profiles. These applications remain promising but require prospective validation before PSMA PET can be incorporated into routine BM imaging algorithms.

### 3.3 Potential clinical role of prostate-specific membrane antigen positron emission tomography in malignant brain tumor imaging

The clinical utility of PSMA-targeted PET imaging in malignant brain tumors should be interpreted alongside established neuroimaging modalities. In gliomas, direct comparisons between PSMA PET and conventional MRI have yielded mixed results. ^68^Ga-PSMA-11 PET has demonstrated higher sensitivity than MRI for distinguishing HGG from LGG, although specificity was comparable between the two modalities (57). A more recent study likewise suggested that PSMA PET–MR and conventional MRI may provide comparable performance for recurrence assessment in selected cases (58). In recurrent GBM, high TBR has been reported (59), and a meta-analysis showed pooled sensitivity and specificity of 98.2% and 91.2%, respectively, for differentiating HGG from LGG (40). Comparative studies have also suggested that PSMA PET may offer advantages over 18F-FDG PET for glioma evaluation (54). Limited comparative evidence also suggests that PSMA PET and amino acid PET may provide partially complementary biological information (60). Nevertheless, MRI and amino acid PET have a substantially more established clinical evidence base in neuro-oncology. Conventional MRI remains the standard imaging platform for diagnosis, treatment planning and longitudinal assessment, while amino acid PET tracers such as ^18^F-FET and ^11^C-methionine (MET) are more extensively validated for selected applications including glioma characterization and post-treatment assessment (61–63). Thus, in gliomas, PSMA PET is best viewed as a complementary modality rather than a replacement for established MRI or amino acid PET approaches.

In BM, PSMA PET has shown detectability across multiple metastatic subtypes. Systematic review evidence supports PSMA-avid lesions in BM from lung cancer, breast cancer, melanoma, thyroid carcinoma, salivary gland tumors, renal cell carcinoma, and prostate cancer (49). A recent prospective study further showed PSMA-positive lesions in 26 of 27 patients with BM from diverse primaries and identified additional lesions not detected on ^18^F-FDG PET/CT in 42% of cases (56). Investigational work has also suggested that super-selective intra-arterial (ssIA) administration can markedly increase lesion uptake compared with intravenous delivery (64). Nonetheless, the evidence remains limited and heterogeneous, and contrast-enhanced MRI remains the standard modality for BM screening and surveillance because of its accessibility, anatomical coverage, and established diagnostic role. Thus, the added value of PSMA PET in BM is likely to lie not in routine lesion detection alone, but in selected contexts such as biological characterization, problem solving when conventional imaging is equivocal, treatment selection for PSMA-directed approaches, and identification of lesions with potential theranostic relevance.

One clinically important application under investigation is the distinction between recurrent tumor and treatment-related change, particularly radiation necrosis. This problem arises frequently in both glioma and BM, where post-treatment enhancement on MRI may reflect viable tumors, therapy-related inflammation, necrosis, or a mixture of these processes. Limited exploratory and case-based evidence suggests that PSMA PET may have value in this setting, as viable-tumor-associated neovasculature may retain PSMA expression, whereas radionecrosis may show a lower targetable vascular signal (29, 65, 66). In glioma, broader PET systematic review data support the usefulness of molecular imaging for distinguishing recurrent tumors from radiation necrosis, although PSMA-specific evidence remains limited and is based on small studies and exploratory reports (29, 65). Similar utility has been reported in selected post-radiotherapy BM cases, where PSMA PET helps support differentiation between active metastasis and necrotic change (49, 66). However, the available evidence remains limited and is based largely on small cohorts and case reports rather than large prospective series. Representative primary studies evaluating PSMA expression and imaging in gliomas and brain metastases are summarized in **Table 1**.

**Table 1:** Representative studies evaluating PSMA expression and imaging in gliomas and BM.

Study [Ref.]	*N*	Tumor type / setting	Study design	PSMA assessment method/ radiotracer	Key findings
Wernicke et al., 2011 (18)	32	GBM	Tissue-based observational study	PSMA IHC	• PSMA was detected predominantly in tumor-associated neovasculature, whereas normal brain vasculature was negative.
Traub-Weidinger et al., 2021 (22)	122	Treatment-naive gliomas	Retrospective observational cohort	PSMA IHC; ^11^C-MET PET correlation	• Vascular PSMA expression was enriched in higher-grade gliomas and was associated with clinicopathological and survival parameters.
Saffar et al., 2018 (36)	72	WHO grade I–IV gliomas	Retrospective tissue-based study	PSMA IHC	• PSMA positivity was more frequent in high-grade than low-grade gliomas, supporting grade-associated vascular expression.
Akgun et al., 2020 (57)	35 analyzed (38 enrolled)	Gliomas	Prospective clinical imaging study	^68^Ga-PSMA PET/MR	• PSMA uptake increased with tumor grade and correlated with pathological features; uptake was particularly useful for distinguishing grade IV tumors.
Van Lith et al., 2023 (30)	14	GBM: newly diagnosed and recurrent	Multicenter inventory study	PSMA PET using multiple tracers; IHC and RNA analysis	• Tumors showed heterogeneous tracer uptake, but uptake did not consistently correlate with overall PSMA expression by IHC or RNA.
Pruis et al., 2025 (31)	12 (7 HGG, 5 BM)	HGG and BM	Prospective PET-MRI-guided multiregional biopsy study	[^68^Ga]Ga-PSMA-11 PET/MRI; IHC	• PSMA was predominantly microvascular; in HGG, PET uptake correlated with microvascular rather than tumor-cell PSMA expression.
Tanjore Ramanathan et al., 2020 (41)	89 BM cases (52 lung, 18 breast, 19 melanoma)	Lung, breast, and melanoma BM	Tissue-based observational study	PSMA IHC	• Vascular PSMA expression varied by primary tumor type and was strongest in lung BM; vascular PSMA in primary lung tumors was associated with faster brain dissemination.
Pei et al., 2023 (42)	7	LCBM	Translational clinical study	^68^Ga-PSMA PET/CT; IHC and molecular/single-cell analyses	• BM showed greater PSMA uptake than matched primary lung tumors, with evidence implicating vascular and brain-microenvironmental cellular compartments.
Van den Broeck et al., 2024 (48)	8	Radioiodine-refractory thyroid carcinoma; intracranial lesions included	Prospective Phase II clinical imaging study	[^18^F]AlF-PSMA-11 PET/CT vs ^18^F-FDG PET/CT; IHC	• Two brain lesions were visualized only on PSMA PET; however, this was a small thyroid-cancer cohort rather than a dedicated BM study.
McLaughlin et al., 2023 (52)	2,763 patients (3,363 scans)	Prostate cancer; incidental intracranial lesions	Retrospective single-center imaging cohort	^68^Ga-PSMA-11 or ^18^F-DCFPyL PET/CT	• PSMA PET identified intraparenchymal and dural metastases but also PSMA-avid benign intracranial lesions, emphasizing limited specificity of uptake alone.
Arslan et al., 2025 (56)	27	BM from diverse primary tumors	Prospective comparative imaging study	^68^Ga-PSMA-11 PET/CT vs ^18^F-FDG PET/CT and MRI	• PSMA-positive BM were detected in 26/27 patients; PSMA PET detected lesions missed by FDG PET in 11 patients, with MRI concordance.
Filizoglu et al., 2021 (66)	1	RCC BM after stereotactic radiosurgery	Case report	^68^Ga-PSMA PET/CT	• Suggested potential utility of PSMA PET/CT in distinguishing recurrent RCC BM from treatment-related radiation changes; evidence remains case-level.

*N* refers to the number of patients, cases, or specimens included in the relevant study cohort, as reported by the original study. Study design is described descriptively to indicate the maturity of the available evidence; no formal evidence-grading framework was applied.

**Figure 3:**
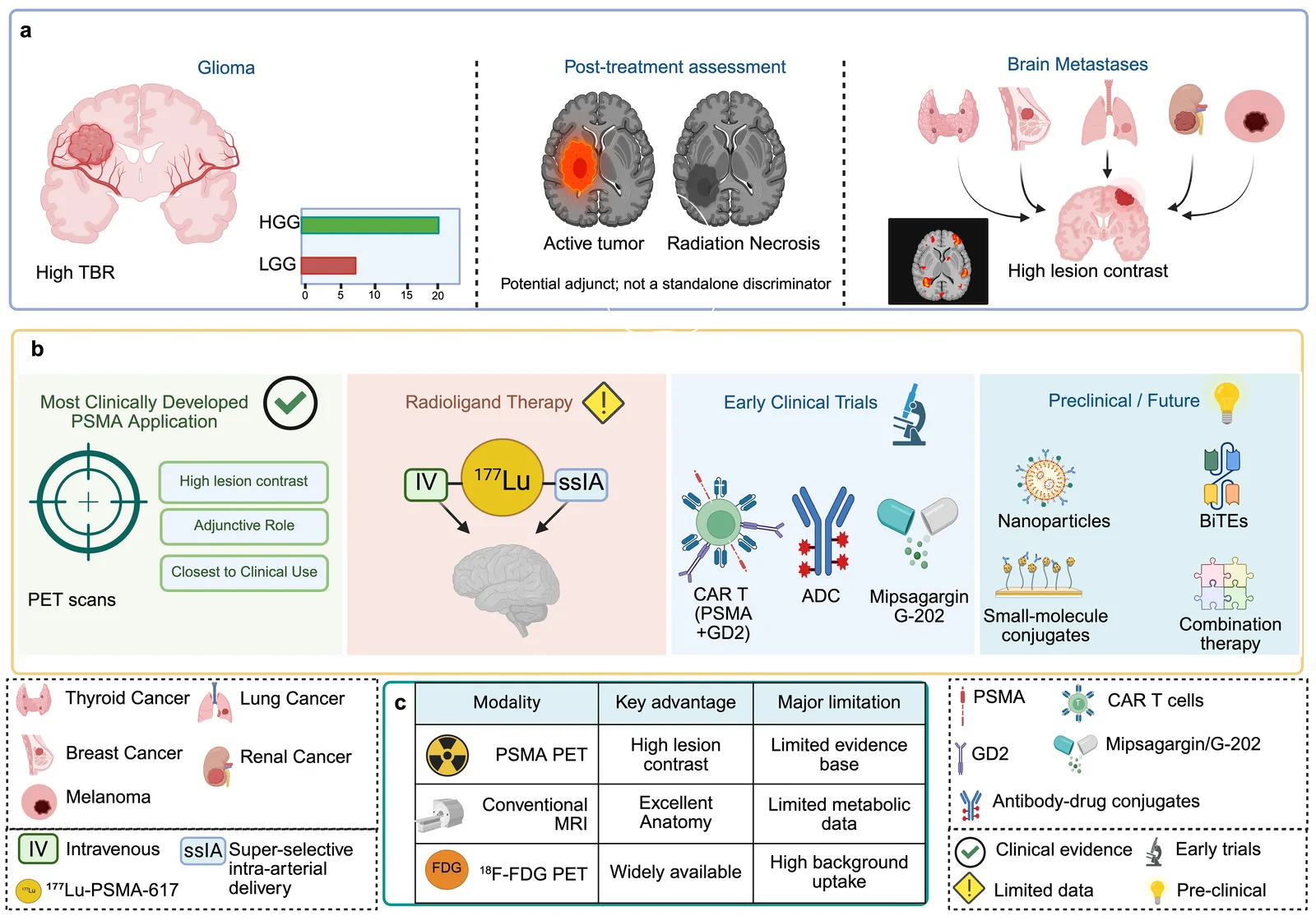
PSMA-targeted diagnostic and therapeutic applications in malignant brain tumors. **(a)** PSMA PET provides high lesion-to-background contrast, with grade-associated uptake in glioma, potential adjunctive utility in post-treatment assessment, and detectable uptake across multiple primary tumor subtypes in brain metastases (BM). **(b)** The therapeutic landscape is organized according to translational maturity, including PSMA PET as the most clinically developed application; radioligand therapy with [^177^Lu]Lu-PSMA-617 and investigational super-selective intra-arterial (ssIA) delivery; early clinical approaches including PSMA- and GD2-directed CAR T cells, antibody-drug conjugates (ADCs), and mipsagargin/G-202; and preclinical platforms (nanoparticles, bispecific T-cell engagers (BiTEs), small-molecule conjugates, and combination strategies). **(c)** This panel summarizes the relative advantages and principal limitations of PSMA PET, conventional magnetic resonance imaging (MRI), and ^18^F-FDG PET. Created with BioRender.com. Pokharel, B. (2026), https://BioRender.com/km4cpj3.

Importantly, false-positive uptake can occur. A recent report described PSMA PET-positive radionecrosis after multimodality treatment for BM, emphasizing that treatment-related changes are not invariably PSMA-negative and that tracer uptake may occasionally reflect inflammatory or reparative vascular processes rather than viable tumor alone (67). For this reason, PSMA PET should not currently be regarded as a standalone discriminator of recurrence versus necrosis. Instead, it is better viewed as a complementary modality whose findings must be interpreted alongside MRI, clinical course, treatment history, and, when necessary, pathological confirmation. The comparative characteristics of PSMA PET relative to established neuroimaging modalities in malignant brain tumors are summarized in **Table 2**, whereas the broader imaging and therapeutic landscape is illustrated (**Figure 3**).

**Table 2:** Comparative roles of prostate-specific membrane antigen positron emission tomography and established imaging modalities in malignant brain tumors.

Imaging modality	Glioma applications	BM applications	Key advantages	Key limitations
^11^C-MET PET	• Established adjunct for post-treatment assessment • May help differentiate recurrence from treatment-related change (63)	• Limited evidence in selected post-treatment settings • May be useful when recurrence versus radionecrosis is uncertain (63)	• Low physiological brain uptake • High lesion contrast • Established clinical utility in neuro-oncology	• Short half-life • Requires on-site cyclotron • Limited availability
^18^F-FDG PET	• Limited intracranial utility because of high physiological cortical uptake • More useful for extracranial staging than for glioma characterization (54)	• Limited sensitivity for intracranial lesion detection • Used more often for systemic staging than detailed BM characterization (49, 56)	• Widely available • Low cost • Useful for extracranial staging	• High background brain uptake • Suboptimal lesion conspicuity • Limited value for intracranial differential diagnosis
^18^F-FET PET	• Used for differentiating HGG from LGG and post-treatment assessment • Broader clinical validation than PSMA PET in glioma imaging (61, 62)	• Limited role • May be used in selected post-treatment BM evaluation • Less established in BM than in glioma (61)	• Low background uptake • Dynamic and quantitative assessment possible • Broader validation in neuro-oncology than PSMA PET	• Requires tracer availability and specialized infrastructure • Not universally available
Conventional MRI (CE-T1, T2-FLAIR, DWI)	• Standard imaging modality for diagnosis • Anatomical delineation and surveillance • Central tool for post-treatment evaluation (57, 58)	• Standard of care for BM screening, diagnosis, and follow-up	• Widely available • Excellent anatomical detail • No ionizing radiation • Standard of care	• Limited specificity for differentiating viable tumor from treatment-related change • Provides limited metabolic information
PSMA PET (^68^Ga/^18^F)	• High uptake in HGG and recurrent lesions • Promising adjunct for glioma grading and selected post-treatment assessment (24–28, 40, 57, 59)	• Detectable uptake reported across several BM subtypes • May help identify lesions of potential theranostic interest (42, 46–49, 52, 53, 55)	• Very low physiological brain background • High lesion-to-background contrast • Potential theranostic relevance	• Evidence base remains limited and heterogeneous • Not guideline-recommended as a first-line neuro-oncology imaging modality • Uptake does not necessarily translate into therapeutic feasibility

The table summarizes representative clinical roles, advantages, and limitations of commonly used or investigated imaging modalities in malignant brain tumors. The listed applications reflect the current evidence base and should not be interpreted as formal guideline recommendations or direct equivalence between modalities.

Overall, current evidence suggests that PSMA-targeted PET is best regarded as a promising complementary imaging modality in malignant brain tumors rather than a replacement for established approaches. However, its position relative to conventional MRI and amino acid PET remains incompletely defined, and its routine clinical role will require direct comparative studies and prospective validation.

## 4 Prostate-specific membrane antigen-targeted therapeutic applications in malignant brain tumors

### 4.1 Radioligand therapy

Among PSMA-targeted strategies, radioligand therapy (RLT) has generated the most clinical interest in malignant brain tumors. Importantly, the therapeutic evidence base remains heterogeneous and preliminary, ranging from individual case reports and small single-arm clinical studies to prospective imaging/dosimetry studies, conference abstracts, and preclinical models. These forms of evidence should not be interpreted as equivalent demonstrations of therapeutic efficacy. However, whether the high diagnostic sensitivity of PSMA PET translates into therapeutic efficacy in brain tumors remains an open question. Published clinical evidence for PSMA-targeted RLT in malignant glioma remains dominated by individual case reports and case-based observations. In recurrent GBM, these reports have demonstrated technical feasibility and described radiographic shrinkage, symptomatic improvements, or short-term disease stabilization in selected patients receiving PSMA-targeted RLT (68–71). However, such observations cannot establish treatment efficacy in the absence of larger prospective therapeutic cohorts. These reports provided proof of concept that intracranial tumors with demonstrable PSMA uptake can be reached by PSMA-targeting radioligands. In some patients, treatment was associated with radiographic shrinkage, symptomatic improvement, or short-term disease stabilization, supporting continued interest in this approach.

At the same time, subsequent dosimetric analyses have highlighted major limitations. In contrast to prostate cancer, where tumor cells themselves often strongly express PSMA and support sustained radioligand retention, brain tumors appear to rely mainly on vascular binding. As a result, the therapeutic effect is likely to depend largely on a crossfire mechanism, whereby radiation emitted from vascular-bound ligands reaches adjacent tumor tissue indirectly rather than through uniform tumor-cell targeting (19, 39). This biology raises concerns about whether intravenous RLT can consistently deliver tumoricidal absorbed doses to malignant brain tumors. Indeed, while a single-patient dosimetry report demonstrated measurable tumor uptake with persistent retention over several days (71), a three-patient clinical dosimetry study in HGG reported low tumor-absorbed doses following [^177^Lu]Lu-PSMA therapy (72). These dosimetric observations characterize radioligand delivery and retention but do not independently establish therapeutic efficacy. A systematic review subsequently summarized 40 reported or institutionally identified cases of PSMA-targeted RLT in non-prostatic cancers, including eight patients with brain tumors, and reported generally low toxicity and encouraging outcomes in selected cases (73). However, the predominantly case-based and heterogeneous nature of these data limits conclusions regarding therapeutic efficacy in malignant brain tumors.

To address these delivery constraints, ssIA administration has been investigated as an alternative delivery strategy. In a single-center, open-label, non-randomized prospective imaging study of malignant primary and secondary brain tumors, intra-arterial delivery of PSMA ligand markedly increased lesion uptake and improved TBR compared with intravenous administration, while dosimetric modeling predicted substantially higher absorbed doses for both beta- and alpha-emitting radionuclides (64). Importantly, this was an imaging and dosimetry study rather than a therapeutic efficacy trial. Thus, the increased uptake and modeled absorbed doses do not establish improved tumor control or survival, and whether enhanced delivery translates into clinical benefit requires therapeutic evaluation. A prospective phase I trial has been registered to evaluate adjuvant [^177^Lu]Lu-PSMA-617 in PSMA-expressing IDH-wildtype gliomas following standard treatment (LU-TARGET; ClinicalTrials.gov identifier: NCT07223034), highlighting continued clinical interest in this approach.

### 4.2 Drug-based therapy

Drug-based PSMA-targeted approaches have also been explored, although evidence in malignant brain tumors remains limited. Antibody-drug conjugates (ADCs) targeting PSMA have shown therapeutic activity in prostate cancer and provide a conceptual framework for selective payload delivery to PSMA-positive tissues (74). In GBM, however, a phase II study of a PSMA-targeted ADC in heavily pretreated patients was reported in conference-abstract form and did not demonstrate clear activity, suggesting that simple extrapolation from prostate cancer may not be sufficient in the CNS setting (75). Challenges likely include limited drug penetration, vascular rather than tumor-cell localization of PSMA, and the biological aggressiveness of recurrent glioma.

A related strategy is the use of PSMA-activated prodrugs. Mipsagargin (G-202), a thapsigargin-based prodrug designed to be activated by PSMA, has been evaluated in recurrent or progressive GBM in a phase II study reported as a conference abstract (76). This approach is mechanistically attractive because it exploits PSMA enzymatic activity within tumor-associated endothelium rather than relying solely on ligand retention. Preliminary findings suggested acceptable tolerability and possible disease stabilization in a subset of patients, but evidence remains preliminary and insufficient to establish a clear therapeutic role.

Small-molecule and RNA-based strategies remain even earlier in development. PSMA-targeted siRNA delivery systems have demonstrated proof of concept in preclinical prostate cancer models, but comparable evidence in malignant brain tumors is lacking (77). Overall, current drug-based PSMA therapies in neuro-oncology remain investigational, and their success will likely depend on improved delivery systems and better alignment between therapeutic design and the vascular biology of PSMA in brain tumors.

### 4.3 Cellular immunotherapy

PSMA has also attracted interest as an immunotherapeutic target. Preclinical prostate cancer studies have shown antitumor activity of PSMA-directed chimeric antigen receptor T cells (CAR T cells) and explored strategies to improve their function in immunosuppressive tumor environments (78, 79). Early clinical translation in prostate cancer has included a phase I study reported in conference-abstract form and a peer-reviewed phase I trial of armored PSMA-targeting CAR T cells, while broader experience has been summarized in a review of CAR T-cell therapy for solid tumors (80–82).

In malignant brain tumors, this strategy is conceptually appealing because adoptive cellular therapy may address some pharmacokinetic limitations faced by systemic radioligands or large-molecule drugs, although challenges related to tumor trafficking, persistence, antigen heterogeneity, and neurotoxicity remain important. A recent single-arm exploratory study of six patients receiving combined PSMA-targeted and disialoganglioside GD2-targeted CAR T cells in refractory or relapsed gliomas reported preliminary evidence of clinical activity, including disease control in all treated patients, a 50% objective response rate, median progression-free survival (PFS) of 9.0 months, median overall survival (OS) of 24.5 months, and no reported immune effector cell-associated neurotoxicity syndrome (ICANS) (83). However, the small sample size, single-arm design, and dual-target treatment strategy preclude attribution of these outcomes specifically to PSMA targeting. A recent review has similarly highlighted the potential of PSMA-directed cellular therapy for glioma management (39). Although these findings are encouraging, they remain early and require validation in larger, controlled studies. Therefore, PSMA-based cellular therapy should currently be viewed as an investigational strategy with translational potential, particularly as part of multi-antigen strategies designed to address tumor heterogeneity.

### 4.4 Novel delivery platforms and emerging conjugates

Beyond cellular therapy, PSMA vascular localization has been explored as a targetable interface for drug-delivery platforms, with several approaches under early investigation in malignant brain tumors. Nanoparticle-based delivery represents another investigational direction. PSMA-targeted nanoparticles have been developed to improve blood–brain tumor barrier penetration and selective drug delivery to intracranial lesions in preclinical BM models (84). More broadly, PSMA-targeted nanoparticle drug delivery has undergone preclinical and early clinical development in non-CNS settings (85). In preclinical BM models, these systems enhanced intracranial accumulation of therapeutic cargo, supporting the feasibility of using PSMA-positive vascular compartments to concentrate therapeutic agents at the tumor interface. Such platforms may be especially relevant in BM, where vascular targeting could help improve local delivery while limiting nonspecific exposure. However, no corresponding clinical evidence in BM is currently available.

Other PSMA-directed immune and drug-conjugate platforms, including bispecific T-cell engagers (BiTEs) and small-molecule drug conjugates, have been investigated primarily in preclinical prostate cancer models and provide conceptual platforms for potential CNS application (86, 87). At present, however, evidence in malignant brain tumors is absent or highly preliminary (**Figure 3b**).

## 5 Limitations, challenges, and future perspectives

Despite encouraging progress, the clinical application of PSMA-targeted strategies in malignant brain tumors remains constrained by several unresolved biological and technical issues. Three foundational assumptions remain incompletely validated: first, that PET tracer uptake quantitatively reflects compartment-specific PSMA protein expression; second, that endothelial PSMA in brain tumors behaves similarly to prostate cancer tumor-cell PSMA with respect to radioligand internalization and retention; and third, that vascular PSMA binding can deliver sufficient crossfire radiation to adjacent tumor parenchyma to achieve a meaningful cytotoxic effect. A further challenge is that high PSMA PET uptake may not reliably predict therapeutic efficacy, particularly in non-prostatic tumors, where early washout and low absorbed dose may limit treatment success despite apparently favorable imaging findings (88).

Addressing these uncertainties will require more rigorous translational study designs. PET–MRI-guided biopsy studies with quantitative immunohistochemistry or autoradiography are needed to define how tracer uptake relates to compartment-specific PSMA expression in malignant brain tumors. Comparative pharmacokinetic studies should clarify whether endothelial PSMA in brain tumors shows radioligand internalization and retention properties comparable to those observed in prostate cancer. In parallel, spatial dosimetry studies integrating histologic mapping with microdosimetric analysis are needed to determine whether radiation delivered from PSMA-positive vessels can reach tumoricidal levels in adjacent tumor tissue. Until such questions are addressed, the therapeutic potential of PSMA-targeted radioligand therapy in brain tumors will remain uncertain. An additional consideration is the physiological neuropeptidase activity of PSMA/GCPII in the CNS (11), which raises a theoretical on-target/off-tumor safety concern for PSMA-directed therapies. The magnitude of this risk is likely to depend on treatment modality and CNS exposure and therefore requires dedicated neurological safety assessment in future clinical studies.

Tumor-specific variability in PSMA expression presents additional challenges. Intertumoral and intratumoral heterogeneity may result in inconsistent uptake patterns, limiting patient selection and response prediction. Interpretation is further complicated by the mismatch between PET imaging, which operates at millimeter-scale resolution, and immunohistochemical validation, which samples tissue at the micrometer scale (31). Multi-omic analysis of low-volume biopsy samples may provide a more rigorous framework for biomarker validation (89). Practical barriers also restrict clinical adoption, including limited tracer availability, specialized production requirements, and the lack of established PSMA imaging pathways for non-prostatic neuro-oncology applications.

Beyond biological validation, analytical tools that improve quantification and standardization may also accelerate clinical translation. In prostate cancer, deep-learning-based lesion segmentation on PSMA PET/CT has shown potential to improve quantitative assessment and treatment monitoring (90). In neuro-oncology, artificial neural networks (ANN)-based MRI response assessment has likewise improved automated tumor quantification, providing a useful precedent for brain tumor imaging workflows (91). More broadly, machine-learning approaches applied to PSMA PET may help standardize lesion detection, quantify heterogeneous tracer uptake, and improve response prediction (92).

Future research should focus on three priorities. First, PSMA detection requires standardization across imaging and tissue-based assays to enable cross-cohort comparison and more uniform diagnostic thresholds; prospective head-to-head studies against current reference standards, including contrast-enhanced MRI and amino acid PET, are essential. Second, therapeutic studies must incorporate rigorous dosimetry and delivery assessment to determine whether meaningful tumor irradiation can be achieved (64, 72). Alternative delivery strategies, such as ssIA administration or blood–brain barrier modulation, as well as alternative radionuclides, warrant systematic evaluation. Third, stronger mechanistic and translational studies are needed, particularly in BM, where the drivers of PSMA expression remain incompletely resolved (42, 49). Integrating radiomics, molecular profiling, and clinical parameters may refine patient selection, while combinations with immunotherapy or radiosensitizers represent plausible translational directions. At present, PSMA PET should be regarded as an adjunct to conventional imaging, and PSMA-targeted therapy should remain investigational.

Overall, PSMA PET appears closer to clinical implementation than PSMA-targeted therapy in malignant brain tumors. Bridging the gap between diagnostic detection and therapeutic translation will require a more precise understanding of compartment-specific PSMA biology, better quantitative validation of imaging biomarkers, and dosimetry-guided therapeutic development across different brain tumor contexts.

## 6 Conclusions

PSMA expression in malignant brain tumors represents a compartment-specific and predominantly vascular feature of the tumor microenvironment, with distinct patterns and regulatory mechanisms across gliomas and BM. These differences provide a strong biological rationale for PSMA-targeted imaging, but they also help explain why therapeutic translation remains challenging. Current evidence supports PSMA PET as a promising complementary imaging biomarker, whereas PSMA-targeted therapy remains investigational because compartment-specific target distribution, heterogeneous expression, delivery barriers, and uncertain dosimetry may limit therapeutic efficacy. Future progress will require standardized imaging and tissue-based detection methods, stronger mechanistic validation, prospective comparative imaging studies, and rigorous dosimetry-guided clinical trials. With these advances, PSMA may evolve from a promising vascular biomarker into a more clinically actionable component of precision neuro-oncology.

## Glossary

2-PMPA: 2-(phosphonomethyl)pentanedioic acid
ADCs: Antibody-drug conjugates
ANN: Artificial neural network
ATF3: Activating transcription factor 3
BiTEs: Bispecific T-cell engagers
BM: Brain metastases
CAR T cells: Chimeric antigen receptor T cells
CE-T1: Contrast-enhanced T1-weighted imaging
CNS: Central nervous system
CT: Computed tomography
CXCL1: C-X-C motif chemokine ligand 1
DWI: Diffusion-weighted imaging
EDN1: Endothelin-1
ER: Endoplasmic reticulum
FDG: Fluorodeoxyglucose
FET: Fluoroethyltyrosine
FOLH1: Folate hydrolase 1
GBM: Glioblastoma
GD2: Disialoganglioside GD2
HGG: High-grade glioma
HIF-1α: Hypoxia-inducible factor 1-alpha
ICAM1: Intercellular adhesion molecule 1
ICANS: Immune effector cell-associated neurotoxicity syndrome
IDH: Isocitrate dehydrogenase
IL6: Interleukin 6
ITGB4: Integrin beta 4
IV: Intravenous
LCBM: Lung cancer brain metastases
LGG: Low-grade glioma
MET: Methionine
MRI: Magnetic resonance imaging
NF-κB: Nuclear factor kappa B
NR4A1: Nuclear receptor subfamily 4 group A member 1
OS: Overall survival
PET: Positron emission tomography
PET/CT: Positron emission tomography/computed tomography
PFS: Progression-free survival
PSMA: Prostate-specific membrane antigen
RCC: Renal cell carcinoma
RLT: Radioligand therapy
SPECT: Single-photon emission computed tomography
SPP1: Secreted phosphoprotein 1
ssIA: Super-selective intra-arterial
SUVmax: Maximum standardized uptake value
T2-FLAIR: T2-weighted fluid-attenuated inversion recovery
TBR: Tumor-to-background ratio
TNF: Tumor necrosis factor
VEGF: Vascular endothelial growth factor
WHO: World Health Organization.
